# CO_2_ Absorption Using Hollow Fiber Membrane Contactors: Introducing pH Swing Absorption (pHSA) to Overcome Purity Limitation

**DOI:** 10.3390/membranes11070496

**Published:** 2021-06-30

**Authors:** Sayali Ramdas Chavan, Patrick Perré, Victor Pozzobon, Julien Lemaire

**Affiliations:** LGPM, CentraleSupélec, Université Paris-Saclay, SFR Condorcet FR CNRS 3417, Centre Européen de Biotechnologie et de Bioéconomie (CEBB), 3 rue des Rouges Terres, 51110 Pomacle, France; sayali-ramdas.chavan@centralesupelec.fr (S.R.C.); patrick.perre@centralesupelec.fr (P.P.); victor.pozzobon@centralesupelec.fr (V.P.)

**Keywords:** pH swing, high purity, membrane contactor, CO_2_ absorption

## Abstract

Recently, membrane contactors have gained more popularity in the field of CO_2_ removal; however, achieving high purity and competitive recovery for poor soluble gas (H_2_, N_2_, or CH_4_) remains elusive. Hence, a novel process for CO_2_ removal from a mixture of gases using hollow fiber membrane contactors is investigated theoretically and experimentally. A theoretical model is constructed to show that the dissolved residual CO_2_ hinders the capacity of the absorbent when it is regenerated. This model, backed up by experimental investigation, proves that achieving a purity > 99% without consuming excessive chemicals or energy remains challenging in a closed-loop system. As a solution, a novel strategy is proposed: the pH Swing Absorption which consists of manipulating the acido–basic equilibrium of CO_2_ in the absorption and desorption stages by injecting moderate acid and base amount. It aims at decreasing CO_2_ residual content in the regenerated absorbent, by converting CO_2_ into its ionic counterparts (${\mathrm{HCO}}_{3}^{-}$ or ${\mathrm{CO}}_{3}^{2-}$) before absorption and improving CO_2_ degassing before desorption. Therefore, this strategy unlocks the theoretical limitation due to equilibrium with CO_2_ residual content in the absorbent and increases considerably the maximum achievable purity. Results also show the dependency of the performance on operating conditions such as total gas pressure and liquid flowrate. For N_2_/CO_2_ mixture, this process achieved a nitrogen purity of 99.97% with a N_2_ recovery rate of 94.13%. Similarly, for H_2_/CO_2_ mixture, a maximum H_2_ purity of 99.96% and recovery rate of 93.96% was obtained using this process. Moreover, the proposed patented process could potentially reduce energy or chemicals consumption.

## 1. Introduction

The last few decades have witnessed an acceleration in climate change research in the carbon dioxide (CO_2_) separation and capture sector. The impact of burning fossil fuels such as coal, oil, and gas towards meeting the world’s energy demands has had a considerable global environmental impact. CO_2_ has a significant impact of all [1]. As a result, many studies are being conducted targeting the technologies for selective CO_2_ removal for post-combustion CO_2_ capture and pre-combustion separation of CO_2_ (biogas or syngas upgrading).

Some of these technologies include cryogenics, absorption column with physical or chemical absorbent, and pressure swing adsorption. These technologies are well established and are also being used commercially. However, the disadvantages of these conventional technologies range from high capital costs, large-space occupancy to energy intensive-regeneration to foaming [2]. More technical challenges include liquid flooding, channeling, absorbent losses, entrainment, and corrosion for which particular attention was given to membrane-based processes.

Membrane contactors are fluid/fluid interacting equipment featuring a hydrophobic polypropylene (PP) membrane that allows non-dispersive contact between fluids. Some of the advantages of membrane contactors include high interfacial area, compactness, easy scale-up, operational flexibility, and independent gas and liquid operation [3,4,5]. However, for new applications, the implementation of membrane contactor technologies on a commercial scale requires an in-depth understanding of mass transfer phenomena and quantitative performance [6]. Membrane contactor technology was first developed for artificial lungs; later in 1985, this technology was applied to CO_2_ absorption [7,8]. Since then, membrane technology evolution and development have targeted specific areas such as post-combustion [9,10,11] and biogas/syngas upgrading [12,13,14].

For biogas or syngas upgrading applications, it is essential to have satisfactory purity, competitive recovery, and reasonable energy consumption to make the process commercially viable. In particular, for applications such as proton exchange membrane fuel cell (PEMFC), hydrogen purity needs to be above 99.97%. Achieving this goal and maintaining the robustness of the process is challenging. The workings of these complex systems and performance of process thus depend upon various parameters such as choice of membrane material, absorbent used, the architecture of the process, and the operating conditions.

### 1.1. Choice of Membrane Material

The use of hybrid dense membrane has been studied widely throughout the literature. A study demonstrated an absorption/desorption process using dense HFMC for biogas upgrading using water as a solvent. This dense membrane could withstand a wide transmembrane pressure corresponding to vacuum desorption due to the dense layer. The given process was able to provide a methane (CH_4_) purity of 98% with about 91% of CH_4_ recovery [15]. In another study, a thin dense layer was added to the porous membrane with CO_2_/CH_4_ selectivity of about 23. This double-step biogas purification process gave a CH_4_ purity of 96% and a methane recovery of 95.6% [16]. E. Chabanon et al. 2011 [17] investigated a dense layer of poly(1-trimethylsilyl-1-propyne) on polypropylene (PP) porous support, which was impermeable to the liquid phase. The presence of a dense layer prevented the wetting of the membrane due to the liquid phase thus reducing resistance to mass transfer, maintaining CO_2_ removal efficiency of 90%. Particular attention should be given to the material of the dense layer used since it governs the mass transfer in the membrane. Adding a dense layer to the porous membrane or using a selective dense membrane may provide intensive solutions, although increasing overall mass transfer resistance [18]. The literature also widely reports the use of hydrophobic polymeric porous membranes [19,20,21,22,23]. Fougerit et al. [24] designed a lab-scale pilot with a porous polypropylene membrane contactor to purify biogas. his pilot could reach a CH_4_ purity of 97.5% and a recovery of 98.7%. Kim et al. [25] also investigated CO_2_/CH_4_ separation using hydrophobic polypropylene contactor with water as absorbent. The methane purity obtained from this process was 97% with a yield of 85%. The hydrophobicity of the membrane poses a non-dispersive environment for gas–liquid absorption allowing operational flexibility. Moreover, the membrane material should be compatible with the absorbent used in the process. PP microporous membrane not only provides mechanical strength (for with standing high transmembrane pressure) it also provides chemical resistance, thermal stability, and strong hydrophobic behavior. Therefore, PP hydrophobic porous membranes was a promising choice.

### 1.2. The Absorbent

Another critical parameter affecting the dynamics of the process is the use of absorbents, broadly categorized into two groups: Physical absorbents and chemical absorbents. Since CO_2_ is acidic in nature, its absorption takes place through acido–basic reactions with a basic absorbent. Amines are the most common and widespread chemical absorbent along with aqueous ammonia [26]. Q. He, et al. 2018 [27] demonstrated a biogas upgrading process using a polypropylene membrane contactor and recovered aqueous ammonia as an absorbent. The highest value of CH_4_ as a retentate in this study was 88% when input biogas flow was 200 mL/min. M. Mavroudi, 2012 [28] showed that amines in higher concentration improve the performance reaching up to 99% of CO_2_ removal from CO_2_/N_2_ mixture. A practice of using aqueous solutions of strong base such as NaOH and KOH has also been reported throughout the literature [28,29,30]. However, the use of excess chemicals and energy requirements for the regeneration of absorbents makes this process less desirable. The physical absorption of gas in a solvent takes place based on Henry’s law. The reaction of CO_2_ with physical absorbents is weak as compared to chemical ones. This feature makes the regeneration of physical solvents more energy efficient. A German engineering company, Ingenieurbüro Buse, developed a biogas upgrading unit using a commercial membrane contactor with water as an absorbent. With a closed-loop operation including absorption and degassing steps, the optimum purity of methane obtained was 98% [13]. Fougerit, et al. [24] managed to obtain a targeted biomethane purity of 97.5% and a corresponding recovery of 98.7% by replacing water with a saline solution of KCl. The purity was maintained along with a high recovery thanks to the methane recycling loop. The use of saline solution induces a “salting-out” effect which augments the recovery of the least soluble gas (CH_4_ in this case).

### 1.3. The Architecture

Apart from the materials, the architecture of the whole process (absorption-degassing) also plays an important part. Connecting two or more membrane contactors in series increases the contact area between the gas and liquid phases. Similarly, using two or more degassing units boost the performance of the system by eliminating dissolved gases. A cascaded stripping configuration developed by [31] not only yields high purity (97%) but also enhanced biomethane recovery (90%). A similar study reported a biomethane purity of 97.34% when operating with one contactor and 99.98% when operating with two contactors connected in series using just water as an absorbent [32]. Unfortunately, the process lacks the regeneration of absorbents, making it less feasible for economic and environmental reasons.

This introduction tells us that the efficiency of the process can be effectively increased by improving the nature of the membrane, choosing reactive absorbents, modifying the process architecture using the membrane contactor, and optimizing the operating conditions for a better recovery rate of the gas of interest. Ultimately, a membrane contactor operation that is paired with the regeneration of absorbents cannot approach a purity of 99%, which seems to be the limitation of this technology. In the present work, an innovative way is proposed to identify the cause behind purity limitation and provide a solution leading to substantial improvements in purity, up to 99.97%. This gas purification process neither consumes excessive chemicals nor generates effluents or liquid waste. The patented process was based on selective CO_2_ absorption into an aqueous salt solution, using hollow fiber membrane contactors highlighting the exploitation of acido–basic equilibrium particularly at the absorption and degassing stages to enhance respective performances. An effect of pH control will be studied closely in forthcoming sections along with the combined effects of gas pressure and liquid flowrate.

## 2. Materials

**Figure 1:**
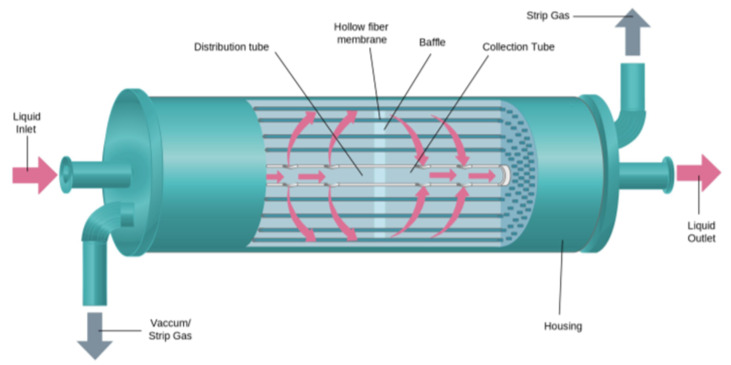
A schematic diagram of Hollow Fiber membrane contactor (adapted from Liqui-Cel^®^ manual) [34].

**Figure 2:**
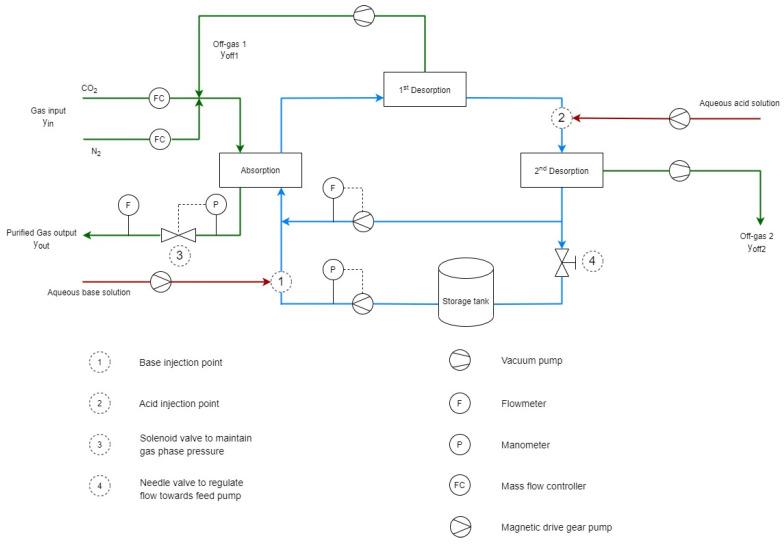
Experimental setup for CO_2_ absorption using series of membrane contactors.

The whole process revolves around the membrane contactor which is shown is Figure 1 and elaborated later in Section 3.1. And the detailed experimental setup used for this study is shown in Figure 2. This setup was designed to track the absorption of CO_2_ from the binary mixture of CO_2_/N_2_ in the salt solution. Pure CO_2_ and N_2_ bottles were provided by Air Liquide with purity > 99.99%. Similarly, for other experimental runs, CH_4_ was also provided by Air Liquide with purity > 99.99%. However, for the hydrogen supply, a hydrogen generator was installed (Swissgas HG series, Torreglia, Italy) for safety reasons. The generator was able to supply the hydrogen at high pressure (up to 10 bars) with a purity > 99.99999%. A synthetic mixture was simulated by mixing CO_2_/N_2_ (35/65 vol%) using Bronkhorst In-flow CTA mass flow controllers. Another mass flow controller (Bronkhorst Low-ΔP-Flow, Montigny Les Cormeilles, France) regulates the gas pressure as well as measures the outlet gas flowrate of HFMC with an accuracy of ±1% of its full-scale capacity.

The salt solution of 1 M concentration was prepared by dissolving industrial grade KCl in filtered reverse osmosis water. Solutions of 1 M HCl and 1 M KOH were prepared in a similar manner. The initial pH of all the solutions was noted to compare future changes. Two pumps (Iwaki magnet drive gear pumps MDG series) were installed to regulate pressure and flowrate inside the absorbent loop. Four pH electrodes (InPro 48XX Mettler Toledo, Viroflay, France) were installed to study the change in pH during the process. A pressure sensor and temperature sensor were installed to measure the pressure drop and temperature of the liquid, respectively. The absorbent temperature was maintained around 291 K ± 1 K with the help of the Huber KISS K6 cooling bath thermostat. Pumps used for injecting the acid and base (Iwaki electromagnetic metering pump EWN-R standard, Marcoussis, France) without affecting the pressure in the loop. The flowrates of acid and base were controlled and adjusted to change pH before absorption and degassing steps.

The input gas, the purified gas, and the offgas were analyzed by micro gas chromatographer (Agilent 490, Les Ulis, France). It is equipped with three molecular sieve columns (10 m Molsieve 5 Å), enabling us to analyze all gases of interest (namely CO_2_, CH_4_, N_2_, and H_2_). The different samples were continuously sent to the micro-GC at a pressure of 200 mbar_g_ through a selection valve (Agilent VICI 6-streams selector valve, Les Ulis, France). Analysis was performed when the purification process reached the steady state. All the streams were continuously analyzed to note even the slightest change in equilibrium. A steady state is said to be achieved when the micro-GC readings show < ±0.5% of absolute fluctuation. The micro-GC was calibrated using standard gas bottles before commencing a new set of experiments. The uncertainty of measurements was ±0.5 *v*/*v*% relatively in the range 5–95%. All gas volume fractions in a sample were normalized so that the sum would be equal to 100%, knowing all gases are quantified and considering that there was no water present in the gas stream. Below 5% *v*/*v*, the relative error remained below 10% until the limit of quantification (50 ppm). Over 90% *v*/*v*, the gas volume fractions are less accurate and were rather estimated from complementary gases content, considered more accurate: (1—Sum of other gases).

Three 2.5”X 8” Liqui-Cel^®^ Extra-flow contactor modules were chosen, one for absorption (X50) and two for desorption (X40) (Provided by Alting, Hoerdt, France). X-40 modules have lower porosity as compared to X-50 modules and as a result, an X-40 module could provide higher mechanical resistance to transmembrane pressures (Table 1). Hence X-40s were selected to perform the degassing step and X-50 modules were used for absorption. The membranes were made up of polypropylene (PP) material and the contact angle of water with PP in the air was 121.6° proving its hydrophobicity [33]. All the runs were performed by ensuring that the membranes were dried before experiments commenced. This was achieved by air drying the membranes overnight between every operation.

## 3. Methodology

### 3.1. Architecture and Design

At the heart of this process is the HFMC, a pseudo crossflow hollow fiber membrane contactor equipped with a central baffle to improve mass transfer. A liquid is introduced on one side of the contactor. It flows outside of the hollow membrane fibers and around the central baffle and exits the contactor through another side. The hollow fibers are connected from one end to the other, allowing open passage to the gas phase (Figure 1).

The process was composed of three membrane contactors connected in series, as shown in Figure 2. Inside a membrane contactor, phases encounter each other owing to the porous membrane structure. Mass transfer between the phases is driven by the gap of equilibrium chemistry of each phase [35]. The gas and liquid phases were passed in a counter-current direction. The gas chassis is independent of the liquid circulation loop. Two mass flow controllers were used to make a synthetic mixture of two gases as a feed. The process gas pressure is regulated with a PID controller connected to the solenoid valve of the outlet mass flow controller. The valve (3) regulates the output flowrate to maintain the pressure in the gas phase. After gas absorption, the saturated absorbent was sent to the two-stage degassing step. In both desorption stages, the liquid was degassed by imparting vacuum inside fibers using vacuum pumps to remove dissolved gas from the liquid. The lean absorbent from degassing step was then circulated back to the absorption contactor through a storage tank, completing a loop. The liquid loop is divided into two sections to regulate flowrate and liquid pressure separately: the feed pump connected to the storage tank is connected to a pressure controller; meanwhile, the circulation pump is connected to a flowrate controller. The valve (4) placed right before the storage tank is used to adjust the short-cut flow to operate the feed pump in adequate conditions (respecting manufacturer’s recommendations).

Base (1) and acid (2) solutions were injected into the absorbent loop before the absorption and desorption steps respectively, with constant flowrate. This addition of ($H^{+}$) and (${\mathrm{OH}}^{-}$) acted as the enhancers of the absorption and desorption process, which is elaborated in the following section.

### 3.2. Theoretical Model

#### 3.2.1. Design and Assumptions

A theoretical model was developed to investigate the contribution of pH variation and imperfect CO_2_ degassing from absorbent in desorption stages on outlet gas purity without considering mass transfer limitation, meaning contactors are large enough compared to gas and liquid flowrates. The present model is based only on acido–basic and gas–liquid absorption equilibria applied at each stage with absorbent recirculation assuming: The absorbent leaving the absorption stage is fully saturated with CO_2_ and the poorly soluble gas *G_a_* to purify (like CH_4_, H_2_, or N_2_). Therefore, the liquid absorbent leaving the contactor is considered in equilibrium with the inlet gas phase, whose composition and pressure are known according to Henry’s law:

(1) $$\left[{\mathrm{CO}}_{2}{}_{\left(aq\right)}\right]=H_{{\mathrm{CO}}_{2}}\times P^{in}\times y_{{\mathrm{CO}}_{2}}^{in}$$

(2) $$\left[G_{a}{}_{\left(aq\right)}\right]=H_{G_{a}}\times P^{in}\times y_{G_{a}}^{in}$$

For each desorption step, total pressure $P_{vac}$ is set by vacuum pumps and the absorbent leaving the contactor is in equilibrium with the gas phase. The partial pressure of CO_2_ and *G_a_* remain in the same proportion as corresponding partial pressures given by Henry’s law from absorbent inlet concentration of CO_2_ (molecular form) and *G_a_* (assuming similar mass transfer resistance between CO_2_ and *G_a_*):

(3) $$P_{{\mathrm{CO}}_{2}}=\left(\frac{C_{{\mathrm{CO}}_{2}}^{in}/H_{{\mathrm{CO}}_{2}}}{C_{{\mathrm{CO}}_{2}}^{in}/H_{{\mathrm{CO}}_{2}}+C_{G_{a}}^{in}/H_{G_{a}}}\right).P_{vac}$$

(4) $$P_{G_{a}}=\left(\frac{C_{G_{a}}^{in}/H_{G_{a}}}{C_{{\mathrm{CO}}_{2}}^{in}/H_{{\mathrm{CO}}_{2}}+C_{G_{a}}^{in}/H_{G_{a}}}\right).P_{vac}$$

The maximum G_a_ purity of output gas $y_{G_{a}}^{\mathrm{out}}$ at the absorption stage is estimated from the minimum CO_2_ partial pressure $P_{{\mathrm{CO}}_{2}}$ out given by Henry’s law from the residual CO_2_ concentration in the inlet absorbent $C_{{\mathrm{CO}}_{2}}$ (imperfectly degassed). Additionally, the output total gas pressure $P^{out}$ is assumed to be equal to input total gas pressure $P^{in}$ (no significant pressure drop):

(5) $$y_{G_{a}}^{out}=\frac{P_{G_{a}}^{out}}{P^{out}}=\frac{1-P_{{\mathrm{CO}}_{2}}^{out}}{P^{in}}\;\leq \;\frac{1-C_{{\mathrm{CO}}_{2}}^{in}/H_{{\mathrm{CO}}_{2}}}{P^{in}}$$

Initially, the absorbent stage was assumed to be fed with fresh absorbent (1 M KCl) with no residual dissolved gases at a given pH. The present model consists of iterative calculation of outlet liquid composition from one stage to the next in the loop until reaching a steady state, knowing its inlet liquid composition. All calculations are based on equilibrium constants of acido–basic and gas–liquid absorption reactions that are involved in this process (Table 2).

To calculate outlet liquid composition, a system of equilibrium and mass conservation equations must be solved. There are many non-linear coupled equations, but the system can be reduced to only one complex equation with only one unknown: the outlet liquid pH. This equation was solved using a simple dichotomy iterative method using Microsoft Excel. Then, all other unknowns corresponding to the outlet liquid composition can be easily deduced from equilibrium and conservation equations.

Finally, the present model, which considers only equilibria can estimate the maximum purity of the poorly soluble gas *G_a_* by calculating the minimum residual CO_2_ concentration (in molecular form) in the inlet absorbent at the absorption stage, depending on operating conditions. It highlighted that purity was mainly limited by residual CO_2_, which remains partially dissolved in the absorbent after degassing stage in the form of hydrogen carbonate and carbonate ions.

#### 3.2.2. Application to Study the Effect of pH Manipulation

**Figure 3:**
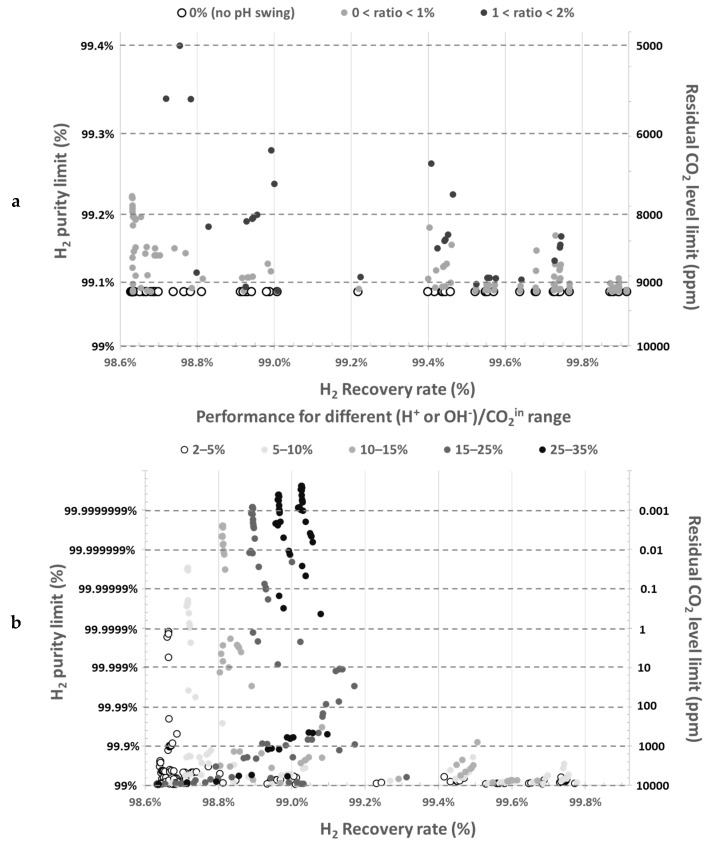
Residual CO_2_ levels in the absorbent as a function of ($H^{+}$ or ${\mathrm{OH}}^{-}$)/CO_2_ ratio. (**a**) Results obtained without pH swing or low ratios and (**b**) Results obtained for larger ratios.

Figure 3 illustrates the effect of acid ($H^{+}$) and base (${\mathrm{OH}}^{-}$) addition on the minimum theoretical residual CO_2_ levels in gas phase and the corresponding maximum H_2_ purity at various operating conditions. Results are depicted as a function of acid or base to inlet CO_2_ ratios. Without acid/base addition or when amounts are too low (Figure 3a), no data point overcomes significantly 99% H_2_ purity. However, Figure 3b highlights that even small acid and base ratios can decrease the residual CO_2_ level to a great extent. In this example, purity over 99%, can be obtained with acid/base molar amount just over 2.5% of the inlet CO_2_ amount (Figure 3b). To overcome residual CO_2_ limit as low as 1 ppm in outlet gas, only 5–10% of acid/base with respect to inlet CO_2_ amount is required. In Figure 3b, the minimum residual CO_2_ level goes below 1 ppb when the acid and base amount correspond to 35% maximum of inlet CO_2_ amount.

This model highlights that purity is mainly limited by residual CO_2_, which remains in the degassed absorbent after the second desorption stage, partially dissolved into hydrogen carbonate and carbonate anions. Figure 3 shows that actual H_2_ purity will remain below 99% (maximum purity corresponding to equilibrium limit) without significant acid/base addition, whatever operating conditions (gas pressure up to 5 bar_g_, vacuum pressure down to 50 mbar_a_ and any gas–liquid flowrate ratio).

**Figure 4:**
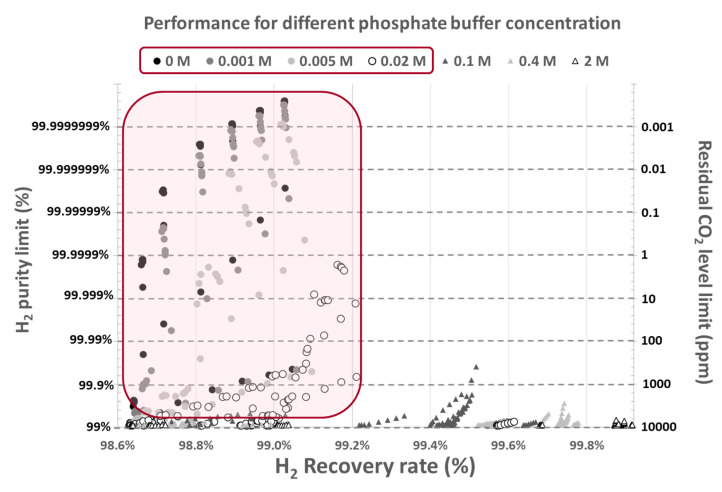
Residual CO_2_ levels in the absorbent as a function of phosphate buffer concentration.

Moreover, the model can further be used to predict the performance of a buffer solution (such as potassium phosphate at different concentrations) as an absorbent. Figure 4 illustrates the effect of phosphate buffer concentration on maximum H_2_ purity and recovery rate that can be achieved (corresponding to the equilibrium limit). This model shows that using buffer solutions increases liquid CO_2_ absorption capacity, and thus increasing the amount of gas that could be treated by the given absorbent. This drastically limits the maximum gas purity that can be reached because of the higher CO_2_ residual amount in the degassed absorbent. In Figure 4, the maximum H_2_ purity remains around 99% when phosphate buffer concentration exceeds 0.2 mol/L, regardless of the operating conditions. Moreover, higher H_2_ purities are always obtained at lower phosphate buffer concentrations in the same operating conditions.

Finally, thanks to this model, it was found that the only way to increase the maximum purity of the poorly soluble gas *G_a_* without consuming much energy, chemicals and increasing capital expenditures was to adjust pH before the absorption and degassing stage. Thus, the maximum purity can be significantly increased by shifting absorbent pH before absorption with the addition of a moderate base amount. Indeed, the residual concentration of dissolved CO_2_ in degassed absorbent can be highly decreased by converting into its anionic forms (${\mathrm{HCO}}_{3}^{-}$ or ${\mathrm{CO}}_{3}^{2-}$). An equal acid amount must be supplied before degassing stage (first or second) to counterbalance the base addition. This serves two purposes: enhancement of CO_2_ degassing by keeping most CO_2_ in molecular form (so keeping a deficient proportion of anionic forms) and stabilizing the overall absorbent pH.

#### 3.2.3. Theoretical Detailed Analysis

Carbon dioxide is physically absorbed in water, obeying Henry’s law. When dissolved in water, some CO_2_ reacts with water, producing carbonate and hydrogen carbonate anions, hydrogen ions, and few intermediate compounds. The formation of $H^{+}$ decreases absorbent pH after the absorption step. Since gas solubility in aqueous solution was proportional to its partial pressure, subjecting the saturated aqueous solution to vacuum will result in degassing of dissolved gas, restoring its pH to a higher value. Interestingly, some CO_2_ will remain dissolved in molecular forms (${\mathrm{CO}}_{2\left(aq\right)}$ or $H_{2}{\mathrm{CO}}_{3}$) or in anionic forms (${\mathrm{HCO}}_{3}^{-}$ or ${\mathrm{CO}}_{3}^{2-}$). This strongly hinders the output gas purity, which is limited by equilibria and the residual CO_2_ amount in molecular form in the degassed liquid.

(6) $${\mathrm{CO}}_{2\left(aq\right)}+H_{2}O\;\dot{↔}{\;H}_{2}{\mathrm{CO}}_{3}\;\dot{↔}\;{\mathrm{HCO}}_{3}^{-}+{\;H}^{+}\;\dot{↔}{\;\mathrm{CO}}_{3}^{2-}+2H^{+}$$

When the base is injected before the absorption contactor into an aqueous absorbent, it dissociates to give ${\mathrm{OH}}^{-}$ anions and corresponding cation (Equation (7)). The base shifts the equilibria at the gas output (liquid input) of the absorption stage to decrease the dissolved residual CO_2_ concentration in molecular form, thus decreasing the remaining CO_2_ concentration in purified gas. Similarly, the injection of acid before the desorption step releases $H^{+}$ into the solution (Equation (8)). These $H^{+}$ ions shift the equilibria of Equation (6) to the left side to promote CO_2_ degassing.

(7) $${\mathrm{KOH}}_{\left(aq\right)}\to {\mathrm{OH}}_{\left(aq\right)}^{-}+K_{\left(aq\right)}^{+}$$

(8) $${\mathrm{HCl}}_{\left(aq\right)}\to H_{\left(aq\right)}^{+}+{\mathrm{Cl}}_{\left(aq\right)}^{-}$$

Thus, the proposed innovative process improves the overall performance in terms of gas purity with relatively low acid and base amounts (less than the absorbed CO_2_ amount). Moreover, acid and base can be efficiently regenerated by bipolar membrane electrodialysis as the absorbent used is a concentrated salt solution [40].

### 3.3. Experimental Operating Conditions

Four series of experiments were designed and performed (Table 3), with CH_4_/CO_2_, N_2_/CO_2,_ and H_2_/CO_2_ gas mixtures with similar sets of operating conditions. A wide variety of operating conditions with different absorbents were tested respecting the limits of process instruments. The main objective behind presenting a comprehensive data set was to showcase the novel technique introduced in Section 3.2. Furthermore, a data set of N_2_/CO_2_ was chosen to study the effect of various operating conditions along with the aforementioned technique to improve purity and yield (Series 1 from Table 3). The dimensionless Henry’s constants of solubility of N_2_, CH_4_, and H_2_ in water at 298.15 K are 1.5 × 10^−2^, 3.4 × 10^−2^, and 1.9 × 10^−2^ respectively [36]. This implies similar absorption behavior among these gases. Therefore, the experiments to examine the effect of operating conditions were first carried out with N_2_/CO_2_ mixture from a safety point of view.

Considering the experimental deviations and error, a few test runs were replicated at similar intervals to check the repeatability of the results. The stability of experimental results in terms of standard deviation was presented in Appendix A.1.

### 3.4. Data Treatment

This study was focused on two key indicators. The first one is the purity $y_{G_{a}}^{out}$ which corresponds to the volume fraction of the gas to purify (%*v*/*v*) at the output of the absorption stage. It is directly measured by micro-GC.

The CO_2_ remaining volume fractions were considered to estimate purity as follows from normalized micro-GC measures.

(9) $$y_{G_{a}}^{out}=1-y_{{\mathrm{CO}}_{2}}^{out}$$

The second indicator is the recovery rate $R_{G_{a}}$ which corresponds to the amount of gas to be purified, recovered at the output of the absorption stage. The recovery rate can be calculated using the inlet and outlet total flowrates and volume fractions of the gas phase. However, gas flowrates are measured less accurately than volume fractions of the gas. Therefore, a formula based on only the volume fraction of the input, output, and second offgas flows was preferred as a viable approximation, considering first offgas is recycled back to input gas:

(10) $$R_{G_{a}}=\frac{y_{G_{a}}^{out}\;}{y_{G_{a}}^{in}}\frac{\;\left(y_{G_{a}}^{in}-y_{G_{a}}^{off2}\right)}{\left(y_{G_{a}}^{out}-y_{G_{a}}^{off2}\right)}$$

## 4. Results and Discussions

### 4.1. Effect of Systematic pH Manipulation

**Figure 5:**
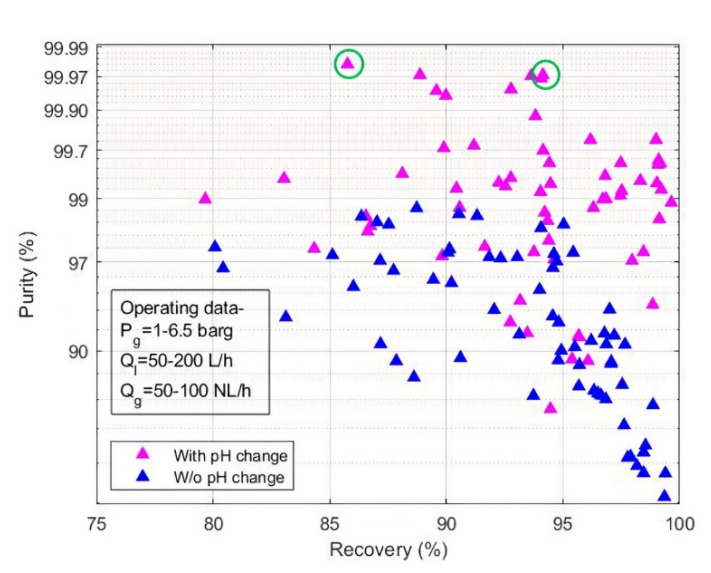
Experimental purity and recovery representing results with and without pH manipulation. (Absorbent: 1 M KCl, 0.5 M K_2_CO_3_ and 0.01 M KHPO_4_, $y_{{\mathrm{CO}}_{2}}^{in}$: 35–40%).

Figure 5 highlights the effect of pH swing on purity and recovery rate of desired gas for all the experiments performed using various operating conditions, absorbents, gaseous mixtures (Table 3). It can be seen from Figure 5 that experimental points subjected to pH swing demonstrated overall better results than the experimental points without pH swing. Some experimental points show low purity even though pH manipulation was performed. This suggests that in these cases, the operating conditions induce significant transfer limitations that degrade purity and recovery rate. That is why, optimal operating conditions such as gas pressure, liquid, and gas flowrates must also be carefully studied. Overall, experimental purity never reached theoretical maximum value as high as shown in Figure 3b, which indicates that mass transfer resistances from gas to liquid always matter. These limitations should therefore be included in the model to predict real performances. For instance, the two best experimental points, circled in green in Figure 5, which reached 99.98% and 99.97% purity, correspond to 99.99998 and 99.9996% theoretical maximum purity in their respective operating conditions. Moreover, they reached 86% and 94% recovery rate, whereas their respective theoretical maximum recovery rate are 99.93%.

### 4.2. Effect of Systematic pH Manipulation

**Figure 6:**
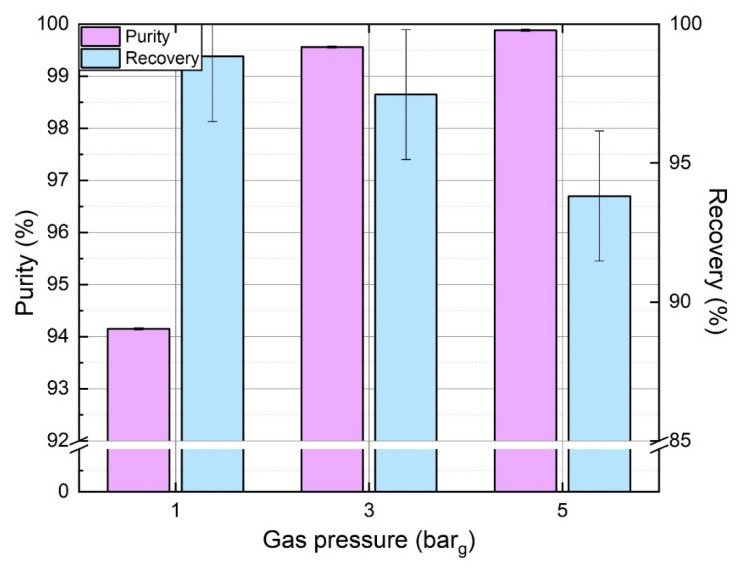
Effect of gas phase pressure on N_2_ purity and recovery rate. (*Q_l_* = 150 L.h^−1^, *Q_g_* = 100 NL h^−1^, $y_{CO2}^{in}$: = 35%, 1 M KCl solution).

At a fixed temperature, absorption of N_2_ and CO_2_ in an aqueous solution highly depends on the operating pressure of the gas phase due to Henry’s law. As the pressure increases, the solubility of gas also increases. Figure 6 shows the effect of gas pressure on purity and recovery rate of N_2_. Experiments were conducted with pH manipulation, where pH before absorption was set at 8 by injecting an alkaline solution at a constant flowrate. Similarly, the corresponding acid is also injected at the same flowrate before the desorption stage, maintaining the pH at 6. It can be seen from Figure 6 that purity can go as high as 99.88 % at 5 bar_g_. A classic tradeoff is further observed during the results with the purity increasing and the recovery rate decreasing. This phenomenon was also supported by the literature [31]. In fact, at higher gas pressure, more N_2_ and CO_2_ are dissolved in the liquid phase resulting in a lower N_2_ recovery rate (an increase of N_2_ loss in the offgas). Meanwhile, N_2_ purity is higher in output gas because more CO_2_ is absorbed relatively. Furthermore, the minimum CO_2_ residual partial pressure (depending on residual CO_2_ concentration in the degassed absorbent) is lower in proportion at higher gas pressure. As a result, operating gas pressure must be high enough to reach high purity, and pH manipulation can be insufficient if gas pressure is too low.

### 4.3. Effect of Liquid Flowrate

**Figure 7:**
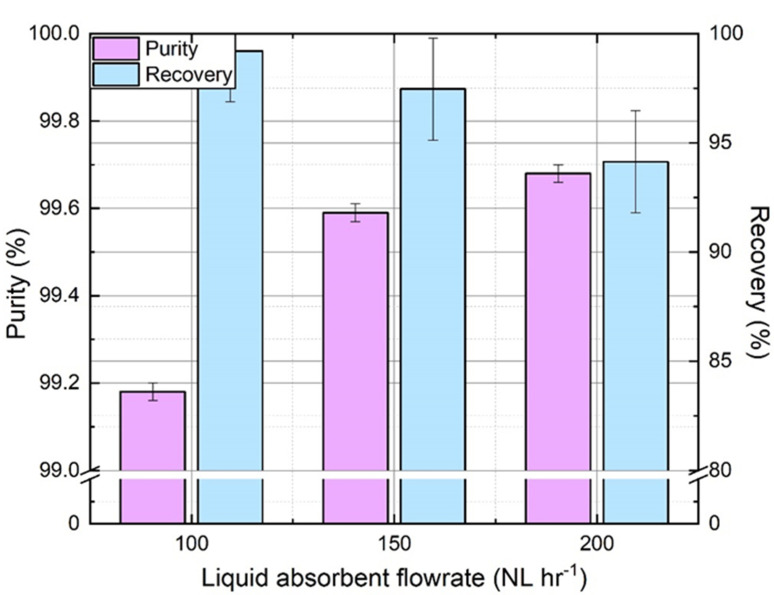
Effect of gas–liquid absorbent flowrate on N_2_ purity and recovery. (*P_g_* = 3 bar_g_, *Q_g_* = 100 NL h^−1^, $y_{{\mathrm{CO}}_{2}}^{in}$= 35%).

Gas concentrations in the liquid phase increase slower along hollow fibers at higher liquid flowrate for a given gas flowrate. The gap between equilibrium and actual gas concentration in the liquid phase (driving force) is higher, which results in a higher amount of gas absorbed. Moreover, a higher liquid flowrate may improve the gas transfer rate at the interface between liquid and membrane by reducing the boundary layer. Consequently, the N_2_ recovery rate is lower (an increase of N_2_ loss in offgas), but purity is higher as more CO_2_ is absorbed. Figure 7 confirms the expected effect of absorbent flowrate on purity recovery tradeoff i.e., as the liquid flowrate increases, N_2_ purity increases in output gas while N_2_ recovery rate decreases. These experiments were performed with a constant pressure of 5 bar_g_ and a pH adjustment of 8 before absorption. An analogous trend of physical absorption was reported in the literature [41,42]. Although the absorption process was favored by an increase of liquid flowrate, the desorption process was negatively affected by the same, mainly due to the lower gas–liquid contact time [21]. Thus, the residual CO_2_ amount in the degassed absorbent was higher at a higher liquid flowrate. Despite this phenomenon, from a global perspective, the performance becomes better with the increase in flowrate. For a given set of operating conditions in the current experimental study, the residual dissolved CO_2_ seems to be the limiting factor to reach high purity. Accordingly, liquid flowrate must be optimized carefully depending on gas flowrate, membrane surface at each stage, and gas transfer rate to achieve high purity.

### 4.4. Magnitude of pH Change

**Figure 8:**
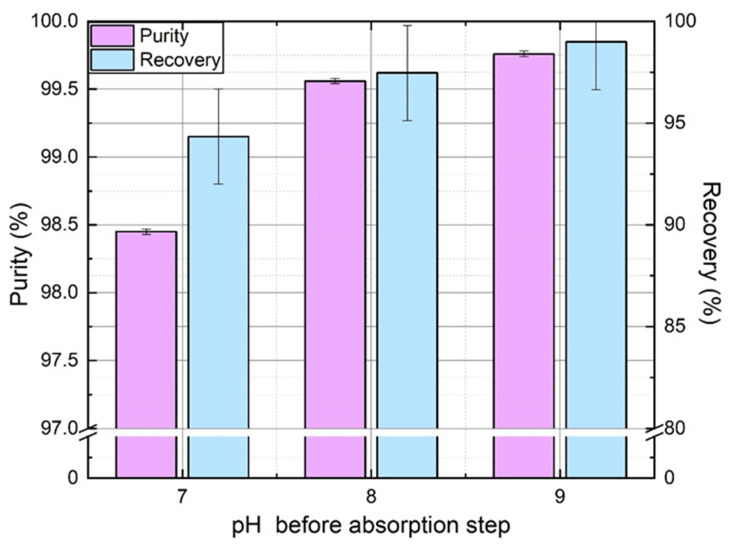
Effect of pH magnitude on N_2_ purity and recovery rate, as observed as the pH value before the absorption step for a constant pH = 6 before the desorption step. (*Q_l_* = 150 Lh^−1^, *P_g_* = 3 bar_g_, *Q_g_* = 100 NL h^−1^, *y^in^*_CO2_ = 35%, 1 M KCl solution).

The effect of pH manipulation was quite evident from Figure 3 in the absence of mass transfer limitations: acid and base injection improve the overall process performance, both in terms of purity and recovery. To confirm this in real experiments, constant flowrate of acid and base was injected ensuring proper mixing with the absorbent. Since the residual dissolved CO_2_ concentration must be the limiting factor at the absorption stage, as stated in Section 3.2, the base flowrate was gradually increased to obtain a pH of 7, 8, and 9 before absorption. The acid flowrate was adjusted to maintain pH 6 before degassing stage. All other operating parameters were kept constant. Figure 8 shows the effect of increasing pH before absorption on nitrogen purity and recovery rate. It is worth mentioning that no tradeoff is observed in this case: both purity and recovery rate are improved by the magnitude of pH swing. As pH before absorption stage must not affect significantly N_2_ absorption, an increase of N_2_ recovery rate must be due to a better recovery in the first degassing stage. It means that more N_2_ is degassed during this stage when pH before desorption (so after absorption) is higher. It can be explained by the lower CO_2_ amount in molecular form to remove at higher pH in the first degassing stage (but similar CO_2_ total amount with its anionic forms). Thus, more N_2_ is removed from absorbent in comparison, so recovered in the first offgas and recycled with input gas at absorption stage.

A key aspect of every process revolves around its feasibility and sustainability. The use of acid-base in the process surely increases the purification of gas with competitive recovery. However, chemical usage raises the question of operational cost. Moreover, acid and base consumption generates salt accumulation in the absorbent, which needs to be partially refreshed and discarded periodically. However, moderate acid and base amount are theoretically sufficient to overcome the 99% purity limit (2% to 5% of CO_2_ amount). However, operating conditions also affect performance as well as mass transfer resistance from gas to liquid. To compare, at $P_{g}\;$= 5 bar_g_, $Q_{g}\;$= 100 NL/h, $Q_{l}\;$= 200 L/h and ${\mathrm{pH}}_{\mathrm{abs}}\;$= 9, the experimental purity reached 99.97%. To maintain a pH of 9 before absorption, 24.5 mL/min of 1 M KOH was required, which corresponds to about 90% of the CO_2_ absorbed amount. Acid and base amount demand are naturally higher than estimations given by the model but still lower than CO_2_ absorbed amount and much lower than the amount used in common chemical absorption processes.

## 5. Conclusions

This work reports a novel CO_2_ separation process using a hollow fiber membrane contactor. This process based on pH swing gives outstanding results in terms of purity and recovery rate. An experimental setup was built using three HFMCs where one membrane contactor was for absorption and the other two for degassing the saturated absorbent. During the theoretical study of this system, it was seen that the residual dissolved CO_2_, due to equilibria with its ionic forms (${\mathrm{HCO}}_{3}^{-}$ or ${\mathrm{CO}}_{3}^{2-}$), restricts the purity that can be achieved with the degassed absorbent. As a solution, the manipulation of acido–basic equilibria by pH swing between absorption and degassing stages was investigated and validated.

The results obtained clearly shows a positive effect of acido–basic manipulation on purity and recovery rate of the least soluble gas, which can be summed up as follows:

- To demonstrate the performance of the process, series of experiments were performed using various operating conditions, which showed us clear advantages of acido–basic manipulation in terms of purity and recovery rate.
- An increase in the gas pressure and liquid flowrate increased the performance in terms of purity, which is consistent with the literature [29,39,40]. However, a classic purity recovery tradeoff was observed when studying the effect of operating conditions.
- An increase in the magnitude of pH change (acid and base injection) improves both purity and recovery rate.
- Even moderate acid and base amount, below CO_2_ amount to absorb (2% to 5%), is sufficient to overcome a theoretical maximum H_2_ purity of 99%.

To sum up, this study introduces a novel technique to improve the CO_2_ separation process using membrane contactors yielding high purity and competitive recovery rate of desired gas. Even if the process involves the use of chemicals in moderate quantities, it can reduce the global operational cost of the absorption processes.

## 6. Patents

A patent was published resulting from this work: Lemaire J., Duval F., Chavan S.R., Pozzobon V., Perré P., Procédé de purification d’un gaz par absorption gaz-liquide. FR2000593 (A1), CentraleSupélec, 22 January 2020.

## Figures and Tables

**Table 1:** Data for X-40 and X-50 2.5” X 8” Liqui-Cel^®^ Extra-flow contactor modules as provided by manufacturers [34].

Parameter	X-40	X-50
Material	Polypropylene (PP)	Polypropylene (PP)
Inner fiber diameter [µm]	200	220
External fiber diameter [µm]	300	300
Porosity [%]	20–25	40–45
Tortuosity	2–3	2–3
Average pore diameter	0.03	0.03

**Table 2:** Equilibrium reactions considered in the present model for H_2_/CO_2_ separation by gas–liquid absorption.

Reactions	Equilibrium Expressions	Equilibrium Constants
${\mathrm{CO}}_{2}{}_{\left(g\right)}\;\overset{H_{{\mathrm{CO}}_{2}}}{↔}\;\;{\mathrm{CO}}_{2}{}_{\left(aq\right)}$	$\left[\;{\mathrm{CO}}_{2}{}_{\left(aq\right)}\right]=H_{{\mathrm{CO}}_{2}}P_{{\mathrm{CO}}_{2}}{}_{\left(g\right)}\;$	$H_{{\mathrm{CO}}_{2}\;}$= 0.034 [36]
$H_{2}{}_{\left(g\right)}\;\overset{H_{H_{2}}}{↔}\;\;H_{2}{}_{\left(aq\right)}$	$\left[\;H_{2}{}_{\left(aq\right)}\right]=H_{H_{2}}P_{H_{2}}{}_{\left(g\right)}\;$	$H_{H_{2}\;}$= 0.00154 [36]
${\mathrm{CO}}_{2}+{\;H}_{2}O\;\overset{K_{1}}{↔}{\;\mathrm{HCO}}_{3}^{-}+{\;H}^{+}$	$K_{1}=\frac{\left[H^{+}\right]\left[{\mathrm{HCO}}_{3}^{-}\right]}{\left[{\mathrm{CO}}_{2}{}_{aq}\right]}$	$pK_{1}$= 6.37 [37]
${\mathrm{HCO}}_{3}^{-}\overset{K_{2}}{↔}{\mathrm{CO}}_{3}^{2-}+{\;H}^{+}$	$K_{2}=\frac{\left[H^{+}\right]\left[{\mathrm{CO}}_{3}^{2-}\right]}{\left[{\mathrm{HCO}}_{3}^{-}\right]}$	$pK_{2}\;$= 10.32 [38]
$H_{2}O\;\overset{K_{3}}{↔}{\;H}^{+}+{\;\mathrm{OH}}^{-}$	$K_{3}=\left[H^{+}\right]\left[{\mathrm{OH}}^{-}\right]$	$pK_{3}\;$= 14 [39]

**Table 3:** Range of operating conditions for given series of experiments.

Series		1	2	3	4
**Inlet gas flowrate**	NL/h	100	100	100	50–100
**Liquid flowrate**	L/h	100–200	50–200	60–200	75–200
**Gas pressure**	bar_g_	1–5	1–5	5	1–6.5
**Transmembrane pressure**	bar_g_	0.25	0.25	0.5	0.25–0.5
**Vacuum pressure**	mbar	50–100	50–100	100	50
**Gaseous mixture**		N_2_/CO_2_	N_2_/CO_2_	CH_4_/CO_2_	H_2_/CO_2_
**Liquid absorbent**		1 M KCl	0.5 M K_2_CO_3,_ 0.01 M KHPO_4_ or 1 M KCl	1 M KCl	1 M KCl

**Figure A1:**
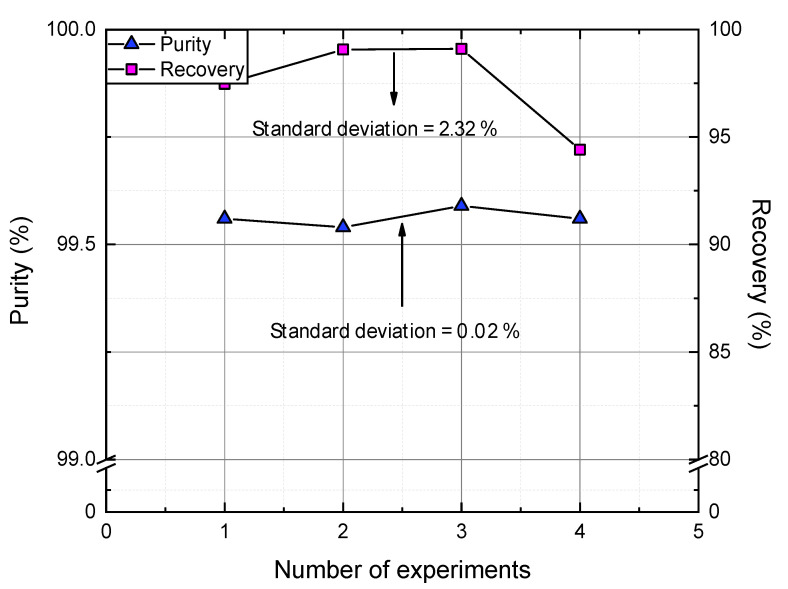
Results of repetition in terms of N_2_ purity and recovery rate (*Q_l_* = 150 L h^−1^, *P_g_* = 3 bar_g_, *Q_g_* = 100 NL h^−1^, $y_{{\mathrm{CO}}_{2}}^{in}$= 35%).

## Nomenclature

$C_{i}.$	Molar concentration of component i in liquid phase
$H_{i}$	Henry’s constant relative to liquid molar concentrations and partial pressure of gaseous component i
N	Number of data points
pK	Acid dissociation constant
$P_{i}$	Partial pressure of the gaseous component i
$P_{g}$	Total gas pressure
$Q_{l}$	Volumetric liquid flowrate
$Q_{g}$	Volumetric gas flowrate
$R_{i}$	Recovery rate of gaseous component i
$\bar{X}$	Mean value
$y_{i}$	Molar fraction of component i in the gas phase
*Greek symbols*	
σ	Standard deviation from mean value
*Subscripts/superscripts*	
||	Absolute value
abs	Relative to absorption stage
$G_{a}$	Poor soluble gas in a gaseous mixture
in	Relative to the inlet flow of the HFMC
out	Relative to the outlet flow of the HFMC
off1	Relative to the outlet of first desorption stage
off2	Relative to the outlet of second desorption stage

## Appendix A

### Appendix A.1. Repeatability and Error Calculation

To measure the effect of environmental factors, human error and experimental deviations, a few experiments were repeated at a similar interval of time, with the same operating conditions. The stability of these experimental results was judged based on its standard deviation from their mean value. As seen in Figure A1, the standard deviation from mean value for purity and recovery rate was 0.02%, and 2.32% respectively.

(A1) $$\sigma =\sqrt{\frac{\sum^{\;}{\left(\left|x_{i}\right|-\bar{\;X}\right)}^{2}}{N}}$$

Standard deviation was calculated using Equation (A1). Where σ was standard deviation, $x_{i}$ was value of purity/recovery, $\bar{X}$ was the mean value and N the number of data points.
